# From Molecule to Material: How Support Changes Heterobimetallic Catalysts in Lactide Polymerization

**DOI:** 10.1002/marc.202500805

**Published:** 2025-12-05

**Authors:** Fan Yang, Yahaya Nasiru, Abdulrahman Adamu Isah, Aimery de Mallmann, Mostafa Taoufik, Régis M. Gauvin, Christophe M. Thomas

**Affiliations:** ^1^ Chimie ParisTech PSL University CNRS Institut de Recherche de Chimie Paris Paris France; ^2^ Laboratoire De Catalyse Polymérisation Procédés et Matériaux (CP2M) CNRS UMR 5128 Univ. Lyon 1 CPE Lyon Université De Lyon Villeurbanne France

**Keywords:** grafted Catalysts, heterometallic Complexes, polylactide, ring opening polymerization

## Abstract

Sustainable production of polylactide demands catalysts that are both recoverable and capable of delivering precise molar mass and stereocontrol. A series of heterobimetallic complexes [(THF)NaFe(O*t*Bu)_3_]_2_, [(THF)_2_KFe(O*t*Bu)_3_]_2_, [KZn(O*t*Bu)_3_]_2_, [(THF)KCu(O*t*Bu)_3_]_∞_ and [(THF)KCo(O*t*Bu)_3_]_2_ was evaluated as precursors for heterogeneous catalysts by grafting onto dehydroxylated silica. All complexes demonstrated activity in the ring‐opening polymerization of lactide. Notably, the silica‐supported [(THF)KFe(O*t*Bu)_2_]_/_SiO_2‐700_ and [(THF)NaFe(O*t*Bu)_2_]_/_SiO_2‐700_ systems exhibited high efficiency, promising recyclability, and afforded predictable molar masses (*M*_n,exp_ close to *M*_n,th_) with narrow dispersities. These findings highlight new opportunities for designing recyclable catalysts for sustainable PLA synthesis.

## Graphical abstract

Silica‐supported heterobimetallic alkoxide complexes were developed as catalysts for lactide ring‐opening polymerization. Grafting iron‐based ate complexes onto SiO_2_‐700 produced well‐defined active sites, enabling controlled polymerization with predictable molar masses, narrow dispersities, and recyclability. Incorporating chain transfer agents further improved precision. This strategy offers a robust platform for sustainable polylactide synthesis and future bio‐based polymer applications.

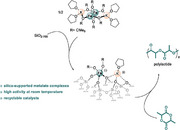

## 1 Introduction

While petrochemical plastics are valued for their versatility, their dependence on finite fossil resources and environmental persistence present significant sustainability challenges [1]. In response, there is growing interest in developing sustainable polymers from biomass‐based monomers [2, 3]. Polylactide (PLA), produced via the ring‐opening polymerization (ROP) of lactide (LA) from annually renewable feedstocks, has emerged as the leading bioplastic [4, 5, 6]. Owing to its high strength, stiffness, clarity, and biocompatibility, PLA is widely used in 3D printing, biomedical devices, and industrial packaging [7].

A wide range of homogeneous catalysts, typically based on metal initiators, exhibit high activity for the ROP of LA [8, 9, 10, 11]. Among these, tin(II) bis(2‐ethylhexanoate) (Sn(Oct)_2_) remains the industrial standard for PLA production [12, 13, 14]. However, the elevated temperatures often required can promote inter‐ and intramolecular transesterification, compromising the control of molecular weight [15]. Furthermore, residual metal impurities from homogeneous catalysts may affect polymer quality and limit applications [16, 17]. In contrast, heterogeneous catalysts offer significant advantages, including straightforward separation, enhanced recyclability, and reduced catalyst contamination in the final product. As a result, the development of well‐defined, supported metal‐based catalysts for the ROP of LA represents a promising strategy to address these challenges and advance sustainable polymer production. Controlled immobilization of metal‐based catalysts is now a well‐established technique [18, 19], enabling the generation of well‐defined surface species from organometallic and coordination complexes. Silica is particularly advantageous as a support due to its well‐understood surface chemistry, which can be precisely tailored to facilitate the grafting process. For example, silica pretreated at 700 °C (SiO_2‐700_) presents isolated, non‐interacting silanol groups as reactive sites, providing an ideal platform for controlled surface organometallic chemistry [18, 19]. This level of control over the nature and uniformity of the surface metal sites is critical, as it directly influences the polymerization performance of the resulting catalysts. In contrast, uncontrolled immobilization can yield a heterogeneous distribution of initiating species with distinct reactivity, leading to poor control over polymer molar mass and stereoselectivity. This strategy has proven highly effective for the ROP of β‐butyrolactone (BBL) using surface‐grafted metal complexes. For instance, grafting [Nd(BH_4_)_3_(THF)_3_] and {Nd[N(SiMe_3_)_2_]_3_} onto silica dehydroxylated at 700 °C enables the conversion of BBL into highly isotactic poly(β‐hydroxybutyrate), thanks to the formation of a single type of surface initiator [20, 21]. Grafting of these species on silica treated at lower temperatures (250 °C and 500 °C) resulted in a lesser control over selectivity, due to the involvement of several types of surface initiating sites. These results underscore the critical role of controlled surface immobilization in tuning the microstructure and properties of the resulting polymers.

Among the various classes of initiators, heterobimetallic catalysts have emerged as particularly promising for the ROP of cyclic esters, often surpassing the performance of their monometallic counterparts [22, 23, 24]. Their enhanced activity is attributed to synergistic intermetallic interactions, which can modulate both Lewis acidity and M─X bond polarity (X = alkyl, alkoxy, carbonate, halide), thereby fine‐tuning the nucleophilicity of the initiating species [25, 26, 27]. Alkali metals are especially attractive partners for classical ROP‐active metals [28] due to their low cost, abundance, and the ready availability of their Li, Na, and K derivatives, enabling systematic tuning of catalytic properties [29]. Notably, the heterobimetallic complex [(THF)NaFe(O*t*Bu)_3_]_2_ has previously demonstrated promising efficiency in lactide polymerization [30], highlighting the potential of such ate‐complexes that combine first‐row transition and alkaline metals for further exploration and development.

**SCHEME 1:**
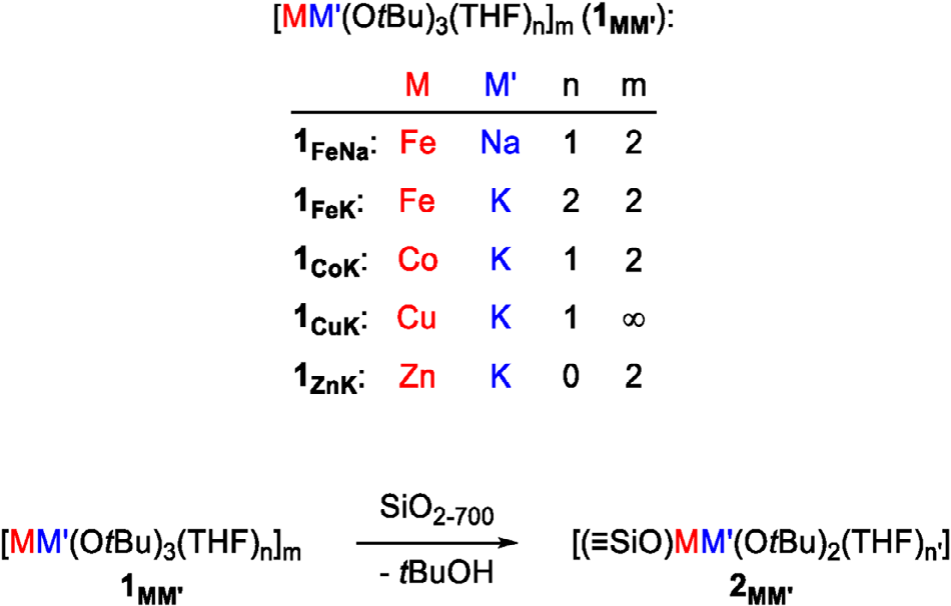
Heterobimetallic 1_MM’_ complexes used in this study and their reaction with SiO_2‐700_.

Building on the recent demonstration by some of us of well‐controlled anchoring of heterobimetallic species onto alumina, we extend this approach to silica supports [31]. In this study, *tert*‐butoxide ate‐complexes (**1_MM’_**), [(THF)NaFe(O*t*Bu)_3_]_2_ (**1_FeNa_**) [32], [(THF)_2_KFe(O*t*Bu)_3_]_2_ (**1_FeK_**) [31], [(THF)KCo(O*t*Bu)_3_]_2_ (**1_CoK_**) [33], [(THF)KCu(O*t*Bu)_3_]_∞_ (**1_CuK_**) [34] and [KZn(O*t*Bu)_3_]_2_ (**1_ZnK_**) [35], were used as initiators for the ROP of PLA, with or without chain transfer agents. Their silica‐grafted analogues (**2_MM’_**) were prepared by reacting the molecular complexes **1_MM’_** with silica dehydroxylated at 700 °C (SiO_2‐700_), enabling the investigation of their performance as immobilized initiators. This comparative study aims to elucidate reactivity trends between the molecular and supported systems, as well as the influence of the transition and the alkali metals. (Scheme 1).

## 2 Result and Discussion

### 2.1 Ring‐Opening Polymerization of *Rac*‐Lactide by Heterobimetallic Complexes

The heterobimetallic complexes were evaluated as initiators for the polymerization of *rac*‐lactide under standardized conditions ([LA]/[Cat] = 100, [LA]_0_ = 1 m in toluene, room temperature) and in the absence of co‐initiators (Table 1).

**TABLE 1:** Polymerization of *rac*‐lactide by heterobimetallic complexes.^a^

Entry	Cat.	Time (min)	Conv. (%)^b^	*M*_n,th_ ^c^ (g/mol)	*M*_n,exp_ ^e^ (g/mol)	*Đ* ^e^	*P*_m_ ^f^
1	**1_FeNa_**	5	90	2200	1600	2.24	0.56
2	**1_FeK_**	5	92	2200	2200	2.29	0.55
3	**1_CoK_**	10	93	2200	4300	1.22	0.59
4	**1_CuK_**	1	87	4200^d^	22000	1.54	0.59
5	**1_ZnK_**	10	92	2300	3800	1.16	0.58

^a^ All reactions were performed with [LA]/[Cat] = 100/1, [LA]_0_ = 1 m at room temperature in toluene.

^b^ Determined by the integration of the ^1^H NMR methine resonances of LA and PLA.

^c^ *M*_n,th_ of polymers calculated from the monomer conversion: ([([LA]_0_/[Cat]) × 144.14 × conversion]/6[Complex]) + MW of end group.

^d^ *M*_n,th_ of polymers calculated from the monomer conversion: ([([LA]_0_/[Cat]) × 144.14 × conversion]/3[Complex]) + MW of end group.

^e^ *M*_n,exp_ and *Đ* determined by SEC‐RI measurements in THF using polystyrene standards and corrected by the Mark‐Houwink parameter for PLA (0.58).

^f^ Probability of *meso* linkage between monomer units, determined by homodecoupled ^1^H NMR spectroscopy.

All complexes exhibited catalytic activity, with **1_CuK_** displaying the highest reactivity, achieving 87% conversion within 1 min (Table 1, Entry 4), significantly outperforming **1_CoK_** and **1_ZnK_**, which required 10 min to reach similar conversions (Table 1, Entries 3 and 5). **1_FeK_** and **1_FeNa_** showed comparable activities (Table 1, Entries 1 and 2). The experimental molecular weights (*M*_n,exp_) are consistent with initiation by three alkoxide groups per metal center. Notably, *M*_n,exp_ values for polymers produced by **1_FeNa_** and **1_FeK_** closely match the theoretical values (*M*_n,th_), with the potassium derivative exhibiting particularly good agreement, likely due to differences in alkali metal ionic radii (Table 1, Entries 1 and 2) [36]. Both **1_FeNa_** and **1_FeK_** yielded PLA with broader dispersities compared to those obtained with **1_CoK_**, **1_CuK_**, and **1_ZnK_**, the latter producing polymers with higher *M*_n,exp_ than *M*_n,th_ (Table 1, Entries 3–5). Notably, the PLA afforded by **1_CuK_** features *M*_n,exp_ that is significantly greater than *M*_n,th_. This indicates that only a fraction of the copper centers are able to initiate polymerization [34, 37]. Microstructural analysis was conducted using homonuclear‐decoupled ^1^H NMR spectroscopy (Figures S13–S19) [38, 39, 40]. Bernoulli statistics were employed to define the relationships between tetrad probabilities and *P*_m_. For instance, in the case of PLA synthesized from *rac*‐lactide with **1_FeNa_**, the homonuclear‐decoupled ^1^H NMR spectrum exhibits well‐resolved, distinct signals in the methyl region (Figure S13). The integration of these signals aligns with the theoretical intensities predicted for a Bernoulli trial process, corresponding to *P*_m_ = 0.56 (Table 1, entry 1). All complexes produce mostly atactic PLA (*P*_m_ = 0.55–0.59, Table 1, Entries 1–5), indicating no significant influence of the metal center on stereoselectivity under these conditions.

To further enhance control over the polymerization process, we investigated the use of chain transfer agents [CA], specifically 2‐propanol and dodecyl mercaptan (DDM) (Table 2) [41, 42]. In both cases, the addition of a chain transfer agent enabled rapid and quantitative monomer conversion within minutes. With 2‐propanol, complexes **1_FeNa_**, **1_FeK_**, **1_CoK_**, and **1_ZnK_** afforded PLA with *M*_n,exp_ in close agreement with *M*_n,th_, demonstrating well‐controlled polymerizations with *M*_n_ proportional to the initial [LA]_0_/[2‐propanol]_0_ ratio (Table 2, entries 1–3 and 7). Notably, the **1_CuK_** system exhibited exceptional activity, achieving complete polymerization of 200 equiv. of *rac*‐LA within 30 s while maintaining good control over molecular weight (Table 2, entry 4). Further decrease of initiator loading led to even narrower dispersities (Table 2, entries 5 and 6). In contrast, the use of DDM yielded high activity and low polydispersity across all complexes, but with less effective control over molecular weight, as evidenced by significant deviations between *M*_n,exp_ and *M*_n,th_ (Table 2, entries 8–12). This discrepancy likely arises from the differing reactivity profiles of M─S vs. M─O bonds, with the lower nucleophilicity of the thiolate group potentially resulting in slower insertion of the metal thiolate (M─S─R) [43], or less efficient M─O*t*Bu/M─SR exchange during initiation.

**TABLE 2:** Polymerization of *rac*‐lactide by heterobimetallic complexes in the presence of a control agent.^a^

Entry	Cat.	Control agent (CA)	[LA]/[Cat] /[CA]	Time (min)	Conv. (%)^b^	*M*_n,th_ ^c^ (g/mol)	*M*_n,exp_ ^d^ (g/mol)	*Đ* ^d^
1	**1_FeNa_**	2‐propanol	100/1/5	1	100	3000	2300	2.02
2	**1_FeK_**	2‐propanol	100/1/5	1	92	2700	2000	2.07
3	**1_CoK_**	2‐propanol	100/1/5	1	100	3000	4800	1.41
4	**1_CuK_**	2‐propanol	200/1/10	0.5	100	3000	2900	1.67
5	**1_CuK_**	2‐propanol	600/1/5	5	100	17000	15000	1.47
6	**1_CuK_**	2‐propanol	800/1/5	5	97	22000	21000	1.46
7	**1_ZnK_**	2‐propanol	100/1/5	0.5	100	3000	4200	1.32
8	**1_FeNa_**	DDM	100/1/5	1	100	3100	8400	1.62
9	**1_FeK_**	DDM	100/1/5	2	92	2900	13700	1.48
10	**1_CoK_**	DDM	100/1/5	0.5	100	3100	35000	1.44
11	**1_CuK_**	DDM	200/1/10	0.5	95	3000	10000	1.55
12	**1_ZnK_**	DDM	100/1/5	7	100	3100	22000	1.43

^a^ All reactions performed with [LA]_0_ = 1 m at room temperature in toluene.

^b^ Determined by the integration of the ^1^H NMR methine resonances of LA and PLA.

^c^ *M*_n,th_ of polymers calculated from the monomer conversion: ([([LA]_0_/[Cat]) × 144.14 × conversion]/[Control agent]) + MW of end group.

^d^ *M*_n,exp_ and *Đ* determined by SEC‐RI measurements in THF using polystyrene standards and corrected by the Mark‐Houwink parameter for PLA (0.58).

### 2.2 Ring‐Opening Polymerization of Racemic Lactide by Grafted Catalysts

As part of our strategy to develop efficient heterogenized initiators for PLA formation, we focused on translating the reactivity of heterobimetallic complexes from homogeneous to supported systems. Achieving this requires the design of well‐defined surface reactive centers, for which silica was selected as the support due to its tunable and well‐characterized surface chemistry. Specifically, we employed non‐porous, high‐surface‐area flame silica (Aerosil 200), which was dehydroxylated under vacuum at 700 °C to yield SiO_2‐700_. This treatment results in a surface populated exclusively by isolated, non‐interacting silanol groups, as confirmed by infrared spectroscopy through the sharp O─H stretching band at 3747 cm^−1^ (Figures S1–S5). Such a surface is ideally suited for controlled grafting via protonolysis with organometallic precursors bearing strongly basic ligands (*e*.*g*., alkyl, amide, or alkoxide), enabling the formation of well‐defined surface organometallic fragments [44, 45, 46].

In direct relevance to the present study, the grafting of alkoxide complexes onto oxide supports has been well established. For instance, [VO(O*t*Bu)_3_] reacts with SiO_2‐700_ to form [(≡SiO)VO(O*t*Bu)_2_] with the release of *tert*‐butanol [47]. Similarly, the heterobimetallic species used in the present study (namely, **1_FeK_**) was recently grafted onto γ‐alumina to generate well‐defined surface species, an approach now extended to silica [31]. Accordingly, the series of ate‐complexes **1_MM’_** (previously used as homogeneous ROP initiators) were reacted with SiO_2‐700_, affording the metal‐decorated materials **2_MM’_** with release of *tert*‐butanol upon grafting. These materials were characterized by IR spectroscopy, combustion analysis for C content, and inductively coupled plasma (ICP) analysis for metal content (Supporting Information). The data confirms retention of the 1:1 M:M’ ratio and an O*t*Bu/M stoichiometry close to 2 (Table S1). Material **2_FeK_** was subjected to further in‐depth characterization. This includes X‐ray absorption spectroscopy (XAS) at the Fe K‐edge: the XANES of **2_FeK_** (Figure S6), shows a pre‐edge at + 1.2 (± 0.2) eV from Fe(0) edge with a maximum normalized intensity of 0.11. This suggests that the Fe sites in the supported complex are in a non‐octahedral coordination with an iron oxidation state of +2 [48, 49]. Electron paramagnetic resonance (EPR) spectroscopy confirmed the absence of Fe(III) and Fe(0) species (Figure S7). High‐resolution transmission electron microscopy (HRTEM) showed no evidence of iron oxide particles, while energy‐dispersive X‐ray spectroscopy (EDX) was in line with a homogeneous distribution of Fe and K centers across the material surface (Figures S8 and S9).

**SCHEME 2:**
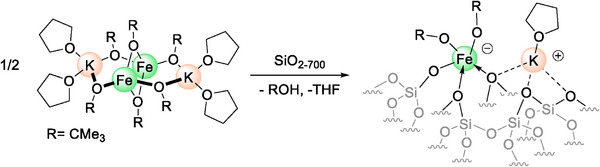
Grafting of **1_FeK_** and proposed structure for **2_FeK_**.

In analogy to the previously reported alumina‐supported iron ate‐complex, we propose that the grafting of **1_FeK_** onto SiO_2‐700_ yields surface sites composed of isolated, formally anionic iron centers coordinated with one surface siloxide and two *tert*‐butoxide ligands, with a potassium cation in the second coordination sphere (Scheme 2).

The catalytic potential of these materials in the ROP of *rac*‐lactide was first evaluated in the absence of a transfer agent. Under these conditions, **2_FeK_** exhibited significant activity, achieving 93% monomer conversion after 4 h at room temperature (Table 3, Entry 1). In comparison, **2_FeNa_** reached only 54% conversion after 9 h and required higher temperatures, further highlighting the performance gap between the corresponding heterobimetallic complexes (Table 3, Entry 2). For both **2_FeK_** and **2_FeNa_**, the experimental molecular weights (*M*_n,exp_) of the resulting polylactide samples closely match the theoretical values (*M*_n,th_), consistent with initiation by two alkoxide groups per metal center. Notably, when comparing polymers obtained from the molecular vs. supported species, the dispersity (*Đ*) significantly decreased, from 2.29 to 1.28 for the potassium derivative and from 2.24 to 1.40 for the sodium derivative analogue, indicating improved control in the polymerization process. This enhanced control is further supported by the linear correlation between molar mass and monomer conversion, with consistently narrow dispersity values throughout the reaction (Figure S20). In this case, first‐order kinetics in monomers were observed, as evidenced by the linear relationship between ln([LA]_0_/[LA]_t_) and time (Figure S21). To evaluate the living nature of the polymerization, a two‐step chain extension experiment was performed using **2_FeK_**. Initially, 200 equivalents of *rac*‐LA were polymerized with 1 equivalent of **2_FeK_**, reaching 93% conversion after 4 h and yielding a polymer with *M*_n,exp_ in close agreement with *M*_n,th_, and a narrow dispersity (*Đ =* 1.28). Upon addition of a second batch of 200 equiv of *rac*‐LA to the same reaction mixture, polymerization proceeded for another 4 h, resulting in a proportional increase in *M*_n,exp_ while maintaining close agreement with *M*_n,th_. These results confirm the presence of a constant population of active chains and demonstrate the living nature of the polymerization (Table S2).

**TABLE 3:** Polymerization of rac‐lactide by grafted initiators.^a^

Entry	Cat.	Temp. (°C)	Time (h)	Conv. (%)^b^	*M*_n,th_ ^c^ (g/mol)	*M*_n,exp_ ^d^ (g/mol)	*Đ* ^d^	*P*_m_ ^e^
1	**2_FeK_**	RT	4	93	13 500	14 000	1.28	0.60
2	**2_FeNa_**	70	9	54	7500	8000	1.40	0.64
3	**2_CoK_**	70	99	0	—	—	—	—
4	**2_CuK_**	70	99	11	—	—	—	—
5	**2_ZnK_**	70	99	0	—	—	—	—

^a^ All reactions performed with [LA]/[Cat] = 200/1, [LA]_0_ = 1 m in toluene.

^b^ Determined by the integration of the ^1^H NMR methine resonances of LA and PLA.

^c^ *M*_n,th_ of polymers calculated from the monomer conversion: ([([LA]_0_/[Cat]) × 144.14 × conversion]/2[Metal]) + MW of end group.

^d^ *M*_n,exp_ and *Đ* determined by SEC‐RI measurements in THF using polystyrene standards, and *M*_n,exp_ corrected by the Mark‐Houwink parameter for PLA (0.58).

^e^ Probability of *meso* linkage between monomer units.

The recyclability of **2_FeK_** was also investigated to assess its practical utility. Following each polymerization cycle, the polymer was removed by filtration, allowing direct recovery and reuse of the catalyst without reactivation. Over three consecutive cycles, the resulting PLA samples consistently exhibited *M*_n,exp_ values in close agreement with *M*_n,th_ and maintained narrow dispersities (Table S3), confirming the robustness and reusability of the supported system. Importantly, immobilization of the metal centers also enhanced stereocontrol. The supported catalysts yielded PLA with slightly improved isotacticity compared to their molecular counterparts: *P*_m_ increased from 0.55 (**1_FeK_**) to 0.60 (**2_FeK_**), and from 0.56 (**1_FeNa_**) to 0.64 (**2_FeNa_**) (Table 3, Entries 1 and 2; Figures S18 and S19). This beneficial effect of surface grafting on stereoinduction has been previously observed in other systems, such as the ROP of BBL and the polymerization of methyl methacrylate [20, 50]. These results highlight the advantages of surface immobilization, not only in enhancing control over molar mass and dispersity, but also in improving stereoselectivity, albeit with a trade‐off in catalytic productivity. End‐group analysis by ^1^H NMR spectroscopy of low‐molecular‐weight PLA obtained from **2_FeK_** revealed termination by a *tert*‐butyl ester and a hydroxyl group, consistent with initiation by *tert*‐butoxide groups (Figure S10). Unexpectedly, the Cu‐based supported material exhibited markedly different behavior from its molecular analogue, yielding only 11% PLA after 4 days, with negligible conversion at shorter reaction times (Table 3, Entry 4). Similarly, grafted Co and Zn species were inactive under the same conditions (Table 3, Entries 3 and 5), highlighting the sensitivity of catalytic performance to both metal identity and support interactions. To further explore the potential of these heterogeneous systems, the influence of chain transfer agents was subsequently investigated.

The 2‐propanol/**2_FeK_** system demonstrated enhanced reactivity compared to its molecular counterpart, producing PLA with narrow dispersity (*Đ* = 1.08) and excellent agreement between experimental and theoretical molecular weights (Table 4, Entry 1). These results suggest near‐quantitative initiation and rapid chain transfer relative to propagation. Under identical conditions, the use of dodecyl mercaptan (DDM) also afforded PLA with a narrow mass distribution (*Đ* = 1.20) and close alignment between experimental and theoretical *M*_n_ values, despite a lower overall conversion (Table 4, Entry 2). Increasing the reaction temperature to 70 °C significantly improved conversion to 95% within 4 h, though at the expense of dispersity, which rose to *Đ* = 1.70, likely due to transesterification processes favored at elevated temperatures (Table 4, Entry 3). Interestingly, the heterogeneous DDM/**2_FeK_** system exhibited different behavior from its homogeneous counterpart (**1_FeK_**). In the supported system, *M*_n,exp_ and *M*_n,th_ remained closely matched, and dispersity values were consistently narrow (Table 4, Entries 2–4 and Table 2, Entry 9). A fivefold excess of DDM was effectively employed, resulting in higher molecular weight PLA, consistent with efficient transfer (Table 4, Entry 4). Similarly, the DDM/**2_FeNa_** system showed improved control over polymerization, further validating the role of surface immobilization in modulating reactivity (Table 4, Entry 5). In contrast, the use of chain transfer agents with the supported Co, Cu, and Zn systems did not yield successful polymerizations under the tested conditions, underscoring the metal‐dependent nature of reactivity in these heterogeneous systems (Table 4, Entries 6–8).

**TABLE 4:** Polymerization of *rac*‐lactide by grafted catalyst in the presence of control agent.^a^

Entry	Cat.	Control agent	[LA]/[Cat]/ [CA]	Temp. (°C)	Time (h)	Conv. (%)^b^	*M*_n,th_ ^c^ (g/mol)	*M*_n,exp_ ^d^ (g/mol)	*Đ* ^d^
1	**2_FeK_**	2‐propanol	200/1/10	RT	2	81	2400	2500	1.08
2	**2_FeK_**	DDM	200/1/10	RT	99	46	1500	1400	1.20
3	**2_FeK_**	DDM	200/1/10	70	4	95	2900	2200	1.70
4	**2_FeK_**	DDM	200/1/5	70	2	87	5200	5600	1.39
5	**2_FeNa_**	DDM	200/1/10	70	4	88	2700	2500	1.57
6	**2_CoK_**	2‐propanol	200/1/10	70	99	21	—	—	—
7	**2_CuK_**	2‐propanol	200/1/10	70	99	0	—	—	—
8	**2_ZnK_**	2‐propanol	200/1/10	70	99	0	—	—	—

^a^ All reactions performed with [LA]/[Cat] = 200/1, [LA]_0_ = 1 m in toluene.

^b^ Determined by the integration of the ^1^H NMR methine resonances of LA and PLA.

^c^ *M*_n,th_ of polymers calculated from the monomer conversion: ([([LA]_0_/[Cat]) × 144.14 × conversion]/[Control agent]) + MW of end group.

^d^ *M*_n,exp_ and *Đ* determined by SEC‐RI measurements in THF using polystyrene standards and *M*_n,exp_ corrected by the Mark‐Houwink parameter for PLA (0.58).

The distinctive performance of the supported iron initiators can be attributed to site isolation resulting from silica grafting. Immobilization of the iron centers on the silica surface leads to the formation of conformationally stable, well‐defined surface species. In contrast, in solution, dynamic intermolecular rearrangements and modifications in the coordination sphere are more likely to occur. This anchoring effect is analogous to complexation with polydentate ligands, where steric protection around the metal center plays a key role in modulating polymerization behavior [39]. While surface immobilization may reduce overall reactivity due to steric hindrance and diffusion limitations (restricting substrate access to the active site), it simultaneously enhances control over key steps such as initiation and monomer insertion. This stabilization of the reactive environment also contributes to improved stereoselectivity, albeit to a lesser extent. Overall, the balance between reduced activity and enhanced control underscores the value of surface‐supported systems in precision polymer synthesis.

## 3 Conclusion

A series of heterobimetallic complexes were successfully immobilized onto silica, yielding well‐defined heterogeneous catalysts for the ring‐opening polymerization of lactide. Among these, the silica‐grafted iron alkoxides [(THF)NaFe(O*t*Bu)_2_]/SiO_2‐700_ and [(THF)KFe(O*t*Bu)_2_]_/_SiO_2‐700_ demonstrated high activity, excellent recoverability, and precise control over molecular weight. The incorporation of chain transfer agents such as 2‐propanol and dodecyl mercaptan further enhanced polymerization control, enabling tailored PLA architectures. This robust and tunable platform opens new avenues for precision polymer synthesis and will be extended to the polymerization of bio‐based monomers, contributing to the advancement of sustainable plastics.

## Conflicts of Interest

The authors declare no conflicts of interest.

## Supporting information




**Supporting File**: marc70166‐sup‐0001‐SuppMat.docx.

## Data Availability

The data that support the findings of this study are available in the supplementary material of this article.
