# Supplementary material for: From Molecule to Material: How Support Changes Heterobimetallic Catalysts in Lactide Polymerization
Source: Macromol Rapid Commun. 2025 Dec 5;47(4):e00805. doi: 10.1002/marc.202500805 (PMC12922730; doi:10.1002/marc.202500805)
Supplement: Supplementary file 1 — Supporting File: marc70166‐sup‐0001‐SuppMat.docx. [file MARC-47-e00805-s001.docx]

**Supporting information**

**From Molecule to Material: How Support Changes Heterobimetallic Catalysts in Lactide Polymerization**

Fan Yang,^[a]^ Yahaya Nasiru,^[b]^ Abdulrahman Adamu Isah,^[b]^ Aimery de Mallmann,^[b]^ Mostafa Taoufik,*^[b]^ Régis M. Gauvin,*^[a]^ and Christophe M. Thomas*^[a]^

**^[a]^** Chimie ParisTech, PSL University, CNRS, Institut de Recherche de Chimie Paris, 75005, Paris, France

**^[b]^** Laboratoire de Catalyse Polymérisation Procédés et Matériaux (CP2M), CNRS UMR 5128, Univ. Lyon 1, CPE Lyon, Université de Lyon, Villeurbanne, F-69616 France

E-mail addresses: mostafa.taoufik@univ-lyon1.fr; regis.gauvin@chimieparistech.psl.eu; christophe.thomas@chimieparistech.psl.eu

**Table of Contents**

[Experimental section 3](#_Toc213109514)

[General polymerization procedure for lactide 4](#_Toc213109515)

[Preparation of materials **2_MM’_** 5](#_Toc213109516)

[Characterization of materials **2_MM’_** 6](#_Toc213109517)

[NMR characterization of polylactide 11](#_Toc213109518)

[Polymerization activity. 21](#_Toc213109519)

[Recyclability of **2_FeK_**. 24](#_Toc213109520)

[Thermal property of PLA. 25](#_Toc213109521)

### Experimental section

All manipulations requiring a dry atmosphere were performed under purified argon by use of standard Schlenk techniques or in a glove box. THF and toluene were freshly distilled from Na-benzophenone prior to use. All solvents were degassed before use unless stated otherwise. Deuterated chloroform-d/99.5% D, Eurisotop was dried over 4 Ǻ molecular sieves. Deuterated benzene-d/99.5% D, Eurisotop was freshly distilled from Na-benzophenone prior to use. Rac-Lactide were recrystallized first in isopropanol, then in toluene, and sublimed twice before use. Isopropyl alcohol from Sigma-Aldrich and dichloromethane from Carlo Erba were trapped two times over CaH_2_ and all were degassed by freeze pump thaw cycles prior to use. All other chemicals were commercially available and were used as received unless otherwise stated.

The NMR spectra were recorded on Bruker Avance-400 and Avance-Neo 500 spectrometers at Chimie ParisTech. ^1^H and ^13^C chemical shifts are reported in ppm versus SiMe_4_ and were determined by reference to the residual solvent peaks for ^1^H and ^13^C NMR. Size exclusion chromatography (SEC) of polymers was performed in THF at 35 °C using an Agilent 1260 Infinity Series GPC (ResiPore 3 μm, 300 x 7.5 mm, 1.0 mL/min, RI (PL-GPC 220) and Light scattering detectors) at Chimie ParisTech. When using the RI detector, the number average molecular masses (*M*_n_) and polydispersity index (*Đ*) of the polymers were calculated with reference to a universal calibration vs. polystyrene standards (limits *M_w_* = 200 to 400,000 g/mol), using a Mark-Houwink correction of 0.58.^[[1]](#endnote-1),^^[[2]](#endnote-2)^ Elemental analyses were conducted under air-free conditions by Mikroanalytisches Labor Pascher, Remagen (Germany). Gas analyses were performed on a Hewlett-Packard 5890 series II gas chromatograph, equipped with a flame ionization detector and an HP PLOT KCl/Al_2_O_3_ column (50 m × 0.32 mm). Diffuse reflectance IR spectra were collected in an air-tight IR cell equipped with CaF_2_. Electron paramagnetic resonance (EPR) spectroscopy was carried out with a Bruker spectrometer Elexsys E500 using X Band (9.4 GHz) radiation at T = 110-120 K, in the Laboratoire de Chimie, ENS Lyon. Samples for EPR were prepared in air-tight quartz tubes loaded within a glove box. For quantitative studies of the paramagnetic phase, double integration of the EPR signal was performed and compared to that of a reference composed of a known amount of vanadyl(IV) sulfate. High resolution transmission electron microcopy (HRTEM) and Energy Dispersive X-ray (EDX) spectroscopy were performed at the Centre Technologique des Microstructures, University Lyon 1 on a JEOL 2100F operating at 200 kV. XAS spectra were acquired at ESRF, Grenoble, France, using BM23 beamline at the iron K-edge in the transmission mode between 7.0 and 8.2 keV. Four scans were recorded at room temperature. Each data set was collected simultaneously with a Fe metal foil and was later aligned according to that reference (first inflection point set at 7112.0 eV). The sample was packed in an argon-filled glovebox within a double air-tight sample holder.

Complexes **1_FeNa_**,^[[3]](#endnote-3)^ **1_FeK_**,^[[4]](#endnote-4)^ **1_CoK,_^[[5]](#endnote-5)^ 1_CuK_**,^[[6]](#endnote-6)^ and **1_ZnK_**^[[7]](#endnote-7)^ were prepared following the corresponding reported methods. SiO_2-700_ was prepared by dehydroxylation of Aerosil 200 (Evonik, 200 m^2^/g) at 700 °C under high vacuum for 12 h.

### General polymerization procedure for lactide

In a glove box, *rac*-lactide (0.5 mmol) was placed in a Schlenk tube. To this tube was added a solution of the corresponding complex (0.05 mmol) in toluene (0.5 mL) and the appropriate control agent. The solution was quickly removed from the glove box and stirred vigorously. After taking a small sample of the crude material with a pipette, conversion was determined by integrating the methine peaks of lactide and PLA in the ^1^H NMR spectrum. The crude mixture was then precipitated with pentane, collected by filtration and dried under vacuum to constant weight.

### Preparation of materials 2_MM’_

In a glovebox, a solution of molecular complex **1_MM’_** in pentane (10 mL) was added to a suspension of SiO_2-700_ (1 g) in a Schlenk flask. The amount of **1_MM’_** was set to achieve a 1 wt% loading in the considered metal (Fe, Co, Cu or Zn). The suspension was stirred at 25 °C for 12 h and the solid was collected by filtration. Analysis of the mother liquor by GC confirmed the release of *t*-BuOH. The resulting solid was washed with fresh *n*-pentane (three times 10 mL) and dried under vacuum (10⁻⁵ mbar), yielding pale yellow (**2_FeNa_**, **2_FeK_**), indigo (**2_CoK_**), green (**2_CuK_**) and white (**2_ZnK_**) materials.

**Table S1.** Elemental analysis of materials **2_MM’_**.

| **2_MM’_** | **M wt%** | **M’ wt%** | **C wt%** | **C/M** | **M’/M** |
| --- | --- | --- | --- | --- | --- |
| **2_FeK_** | 0.95 | 0.7 | 2.9 | 12.8 | 1.03 |
| **2_FeNa_** | 1.16 | 0.42 | 2.2 | 8.8 | 0.87 |
| **2_CoK_** | 1.01 | 0.78 | 2.65 | 12.9 | 1.16 |
| **2_CuK_** | 0.58 | 0.92 | 2.81 | 25.7 | 2.58 |
| **2_ZnK_** | 0.97 | 0.66 | 1.99 | 11.2 | 1.14 |

### Characterization of materials 2_MM’_


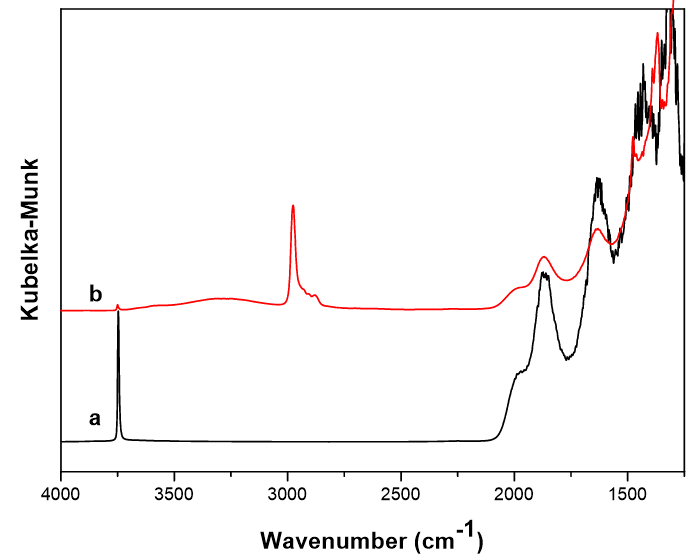


**Figure S1** DRIFT spectra of (a) SiO_2-700_ and (b) **2_FeNa_**.

**Figure S2** DRIFT spectra of (a) SiO_2-700_ and (b) **2_FeK_**.

**Figure S3** DRIFT spectra of (a) SiO_2-700_ and (b) **2_CoK_**.


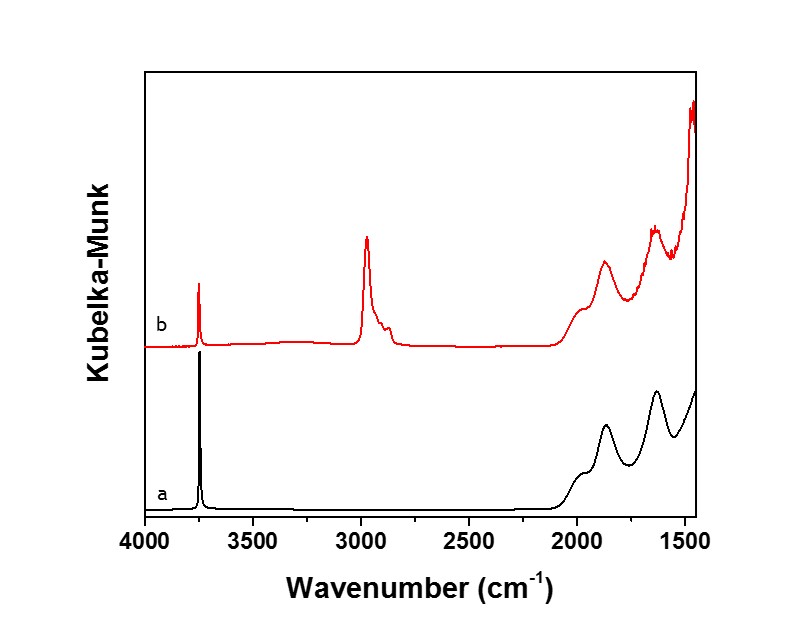


**Figure S4** DRIFT spectra of (a) SiO_2-700_ and (b) **2_CuK_**.


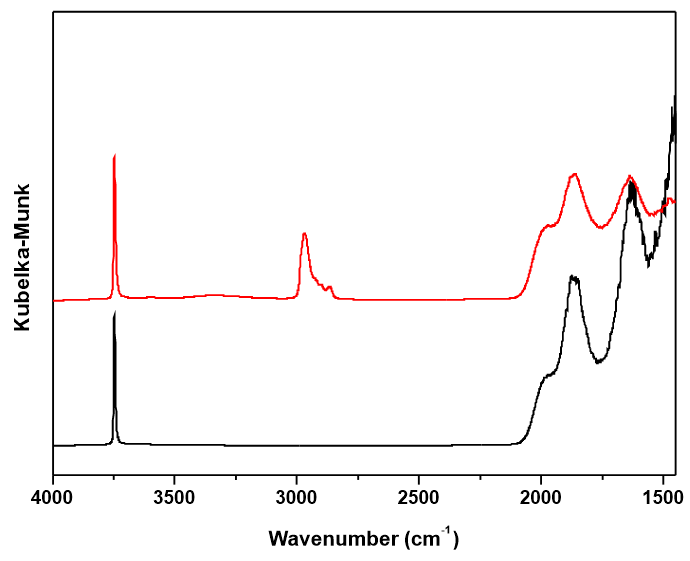


**Figure S5** DRIFT spectra of (a) SiO_2-700_ and (b) **2_ZnK_**.

**Figure S6** XANES of **2_FeK_** (red) and spectrum of an iron foil shown for comparison (dashed green line).

**Figure S7** EPR spectra of **(a) 2_FeK_** (with less than 0.1 % Fe(III)/Fe(total); and **(b)** a V(IV) standard (the vertical scale of this spectrum was divided by 200, for a same metal molecular content).

*
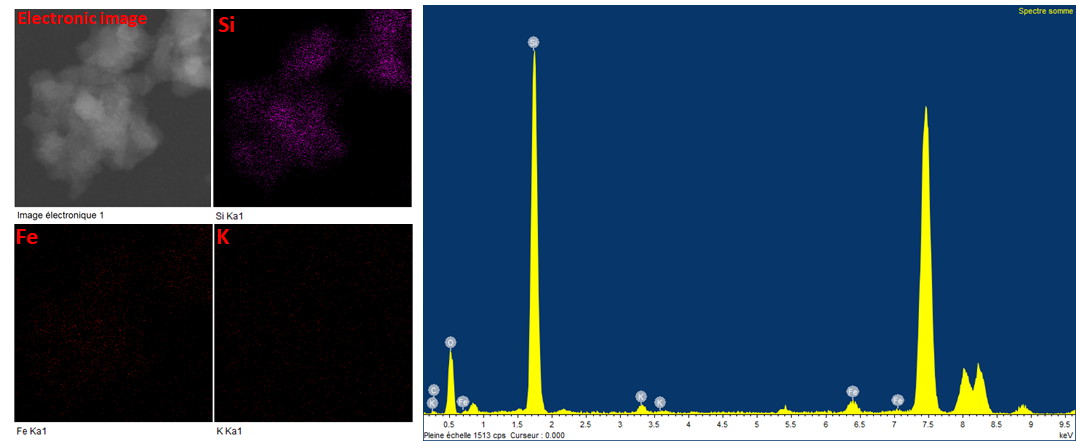
*

**Figure S8** EDX mapping (left) and EDX spectrum (right) of **2_FeK_**.

*
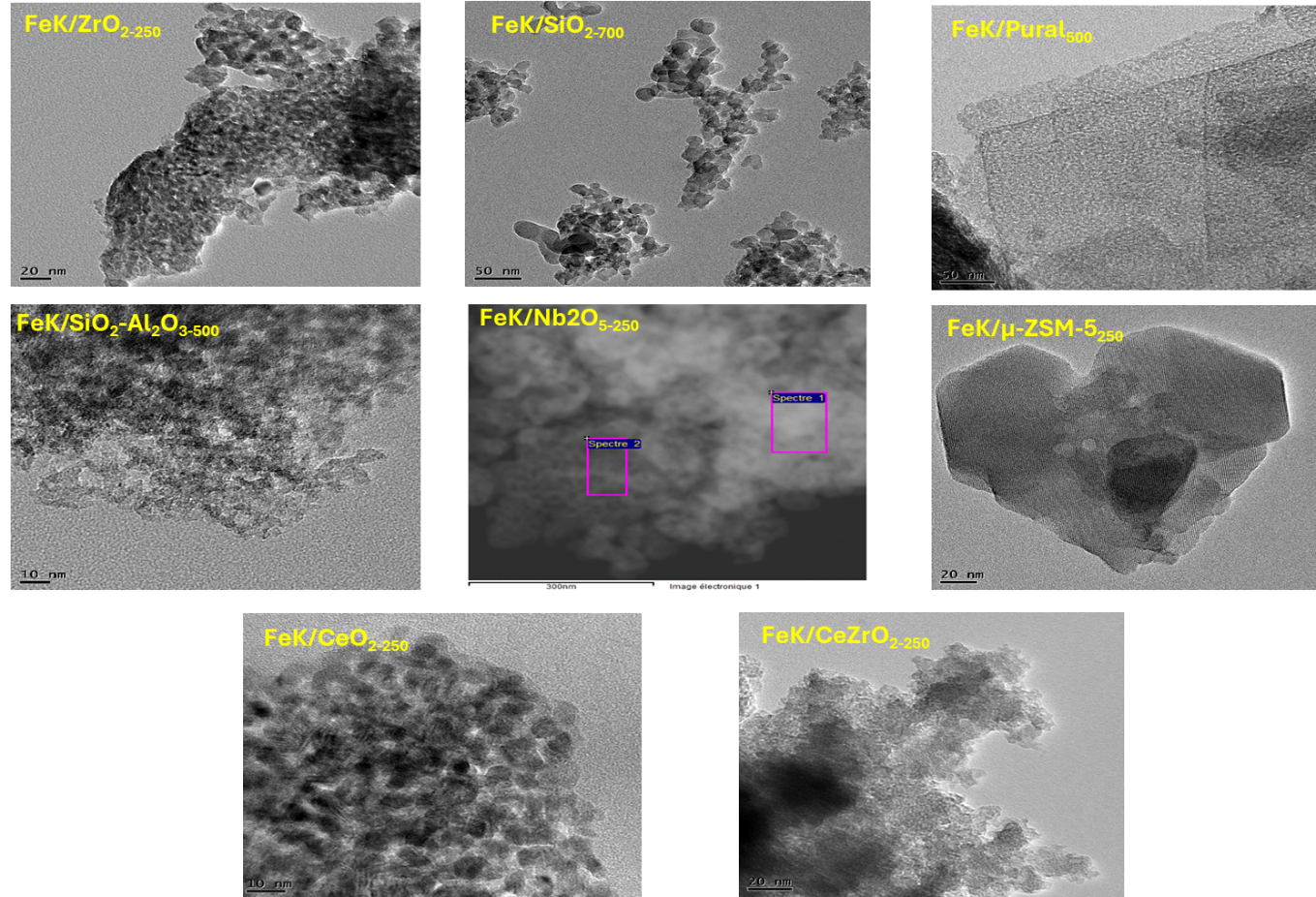
*

**Figure S9** HRTEM image of **2_FeK_**

### NMR characterization of polylactide

**b**

**c**

**a**

**d**

**b**

**b**

**c**

**e**

**c**


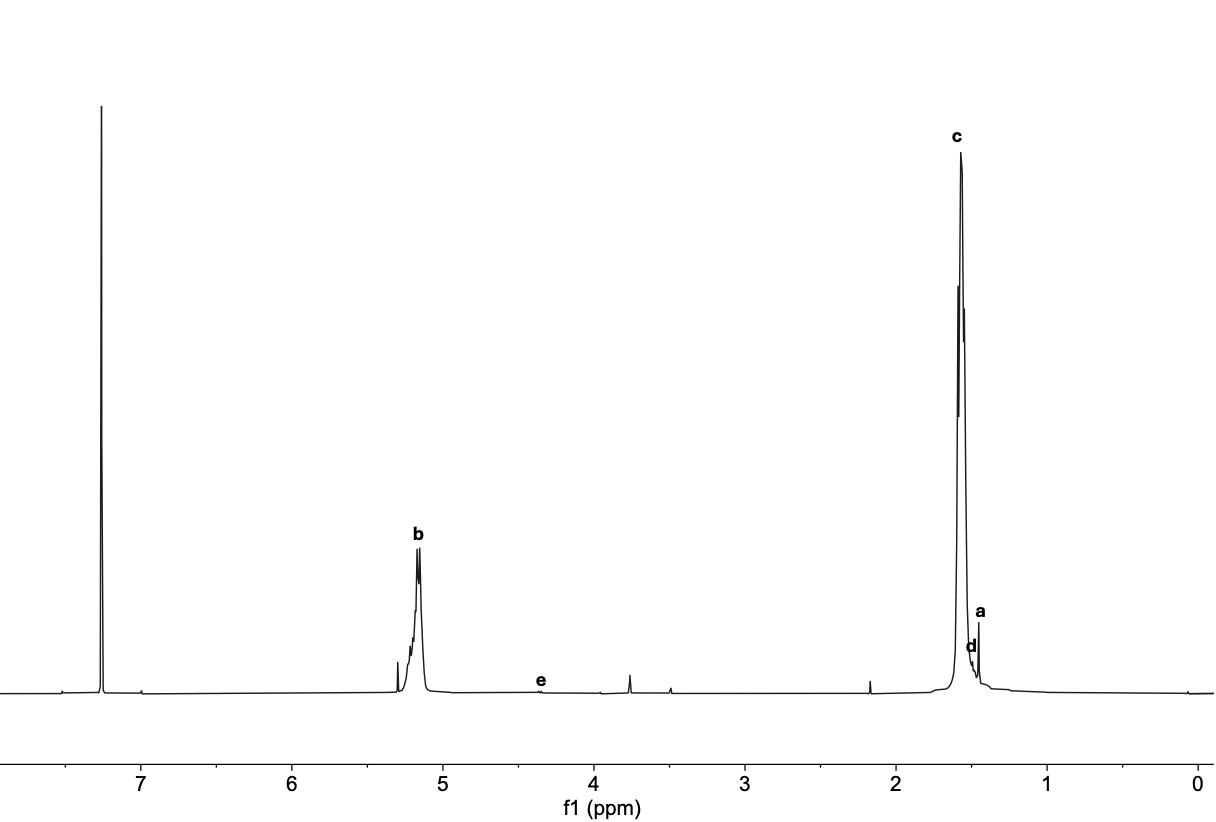


**Figure S10 ^1^**H NMR spectrum of PLA in CDCl_3_ (Table 4, entry 1).

**Figure S11 ^1^**H NMR spectrum of PLA in CDCl_3_ (Table 4, entry 6).


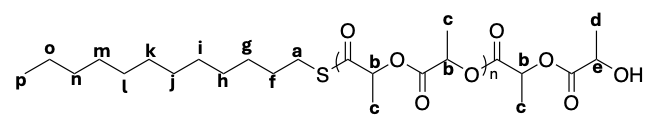


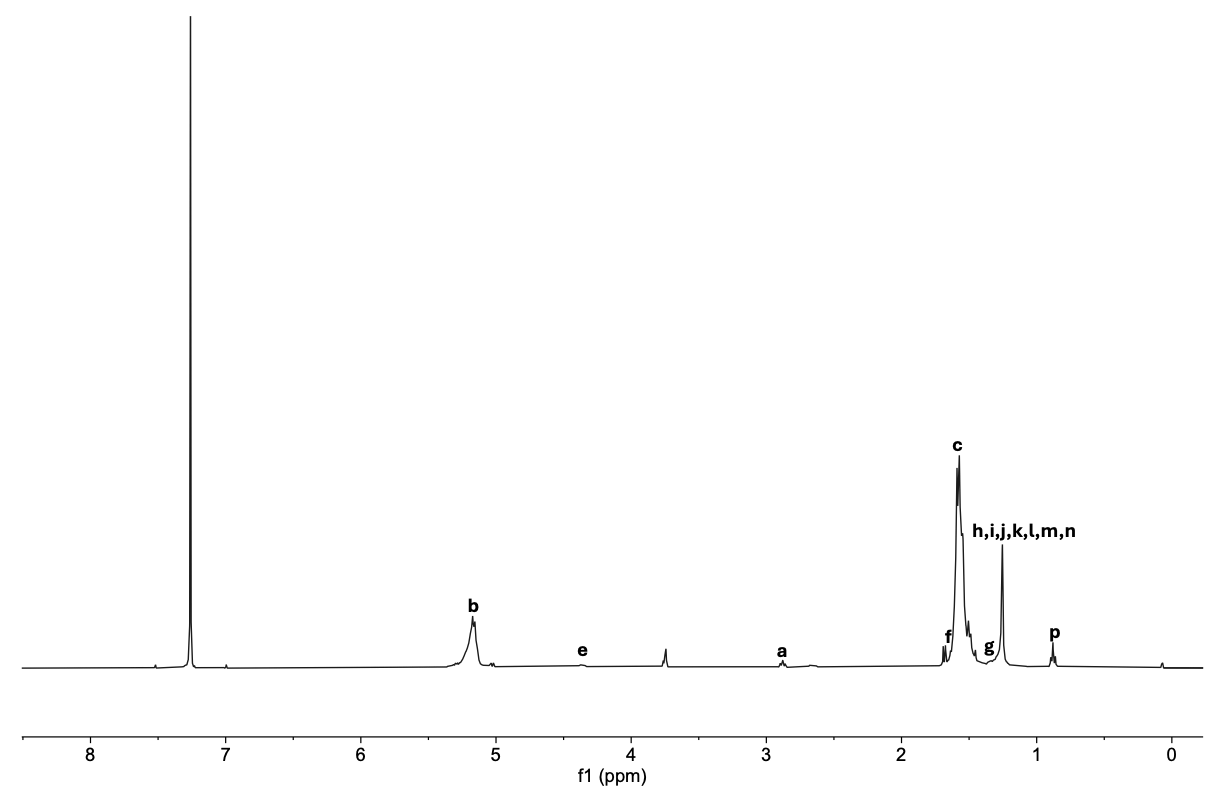


**Figure S12 ^1^**H NMR spectrum of PLA in CDCl_3_ (Table 4, entry 7).


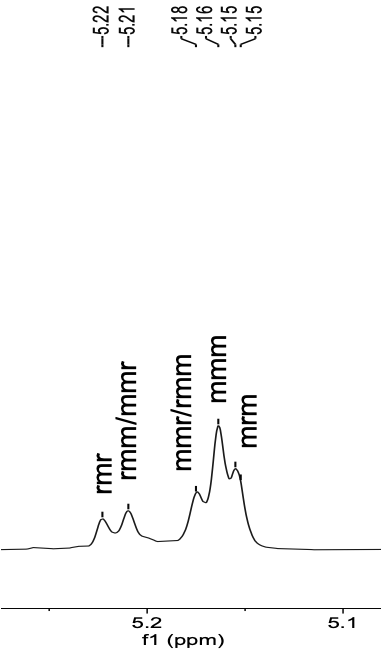


**Figure S13** Methine region of the homodecoupled ^1^H NMR spectrum (500 MHz, CDCl_3_) of a PLA prepared by polymerization of rac-LA with **1_FeNa_**.


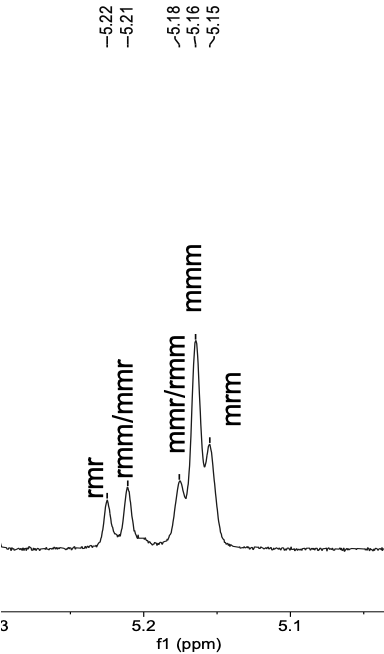


**Figure S14** Methine region of the homodecoupled ^1^H NMR spectrum (500 MHz, CDCl_3_) of a PLA prepared by polymerization of rac-LA with **1_FeK_**.


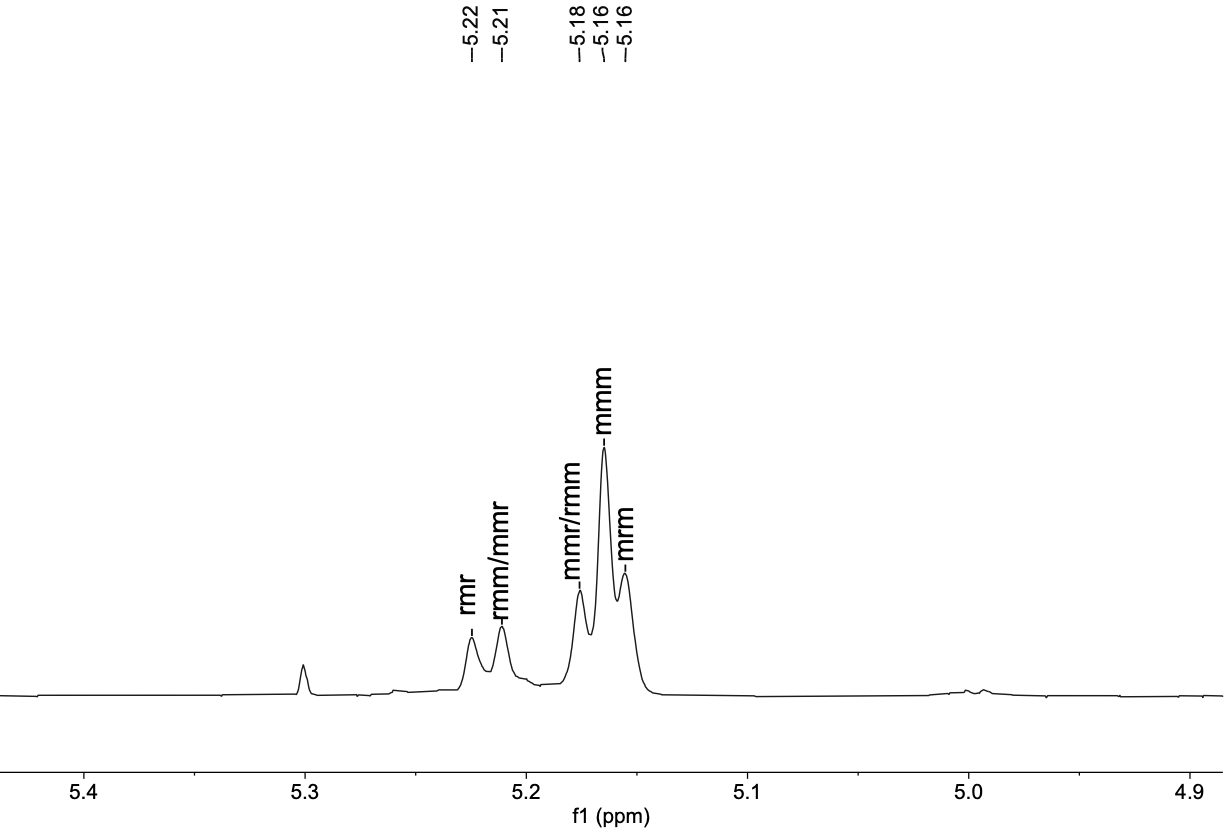


**Figure S15** Methine region of the homodecoupled ^1^H NMR spectrum (500 MHz, CDCl_3_) of a PLA prepared by polymerization of rac-LA with **1_ZnK_**.


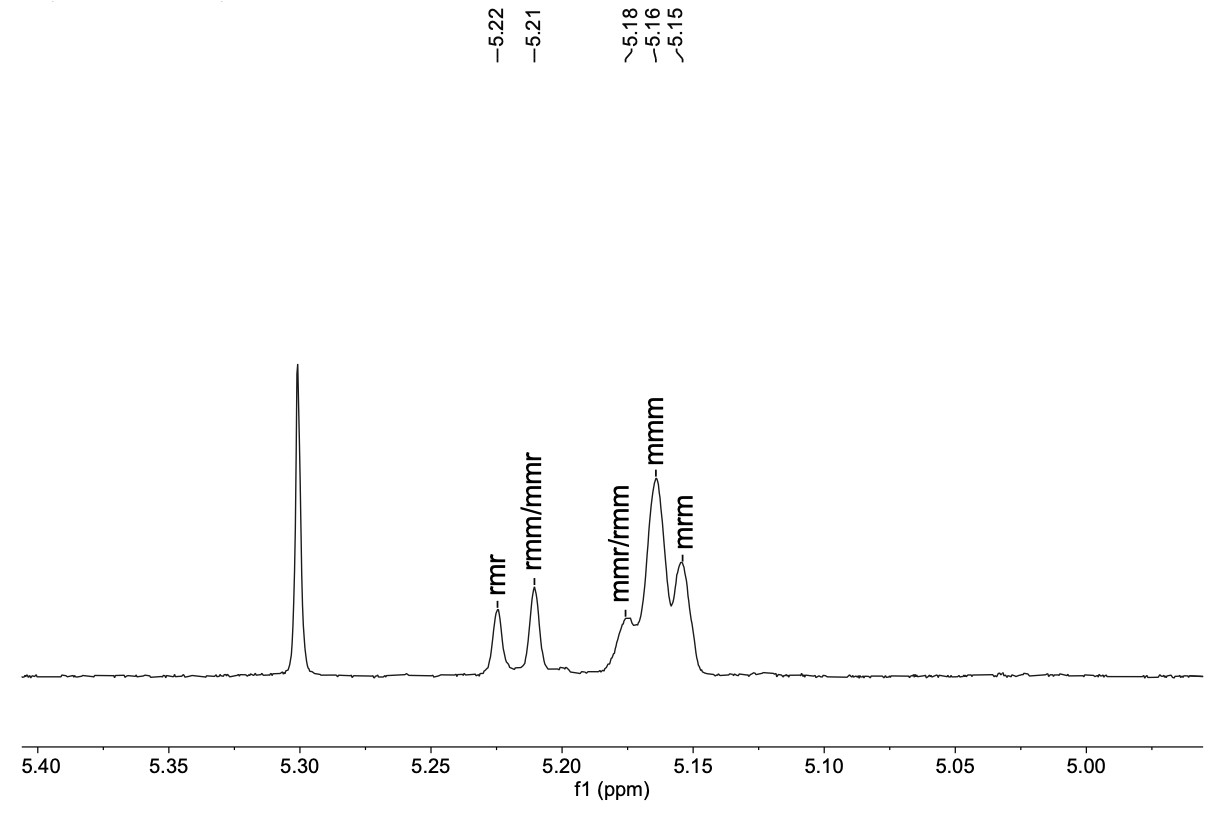


**Figure S16** Methine region of the homodecoupled ^1^H NMR spectrum (500 MHz, CDCl_3_) of a PLA prepared by polymerization of rac-LA with **1_CuK_**.


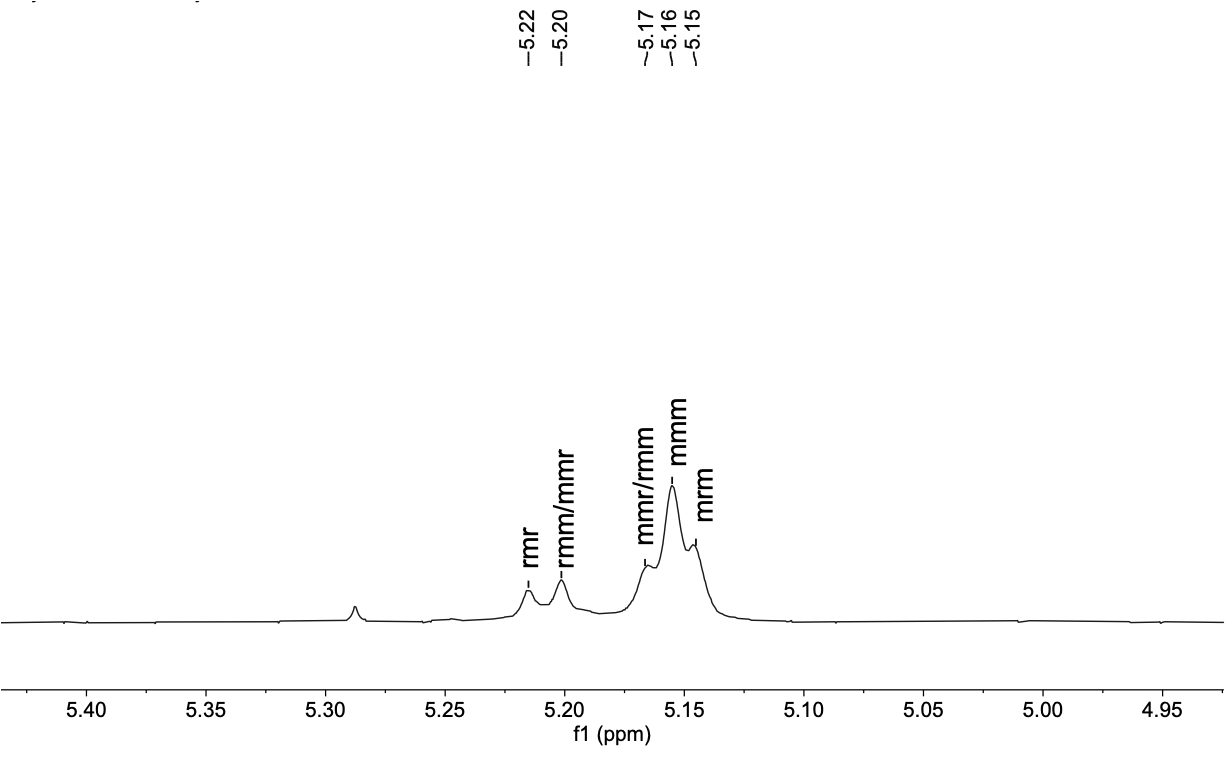


**Figure S17** Methine region of the homodecoupled ^1^H NMR spectrum (500 MHz, CDCl_3_) of a PLA prepared by polymerization of rac-LA with **1_CoK_**.


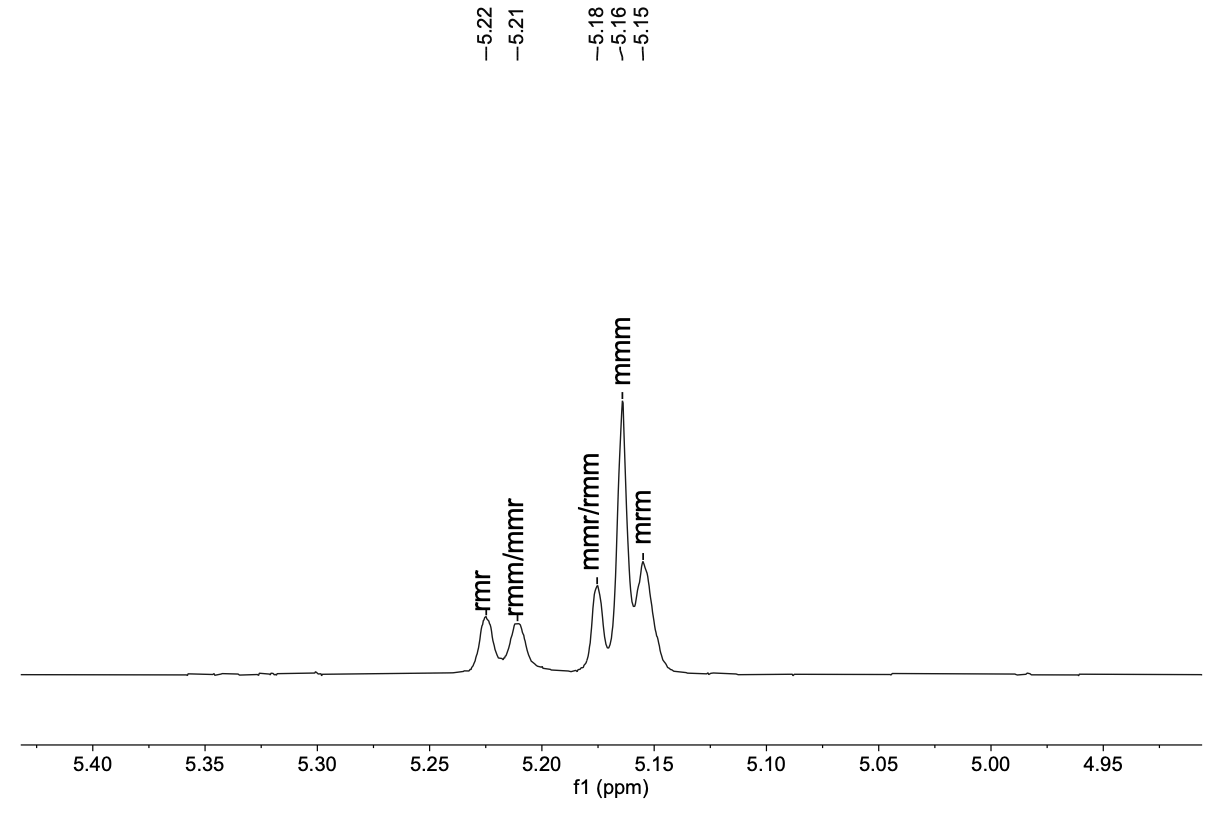


**Figure S18** Methine region of the homodecoupled ^1^H NMR spectrum (500 MHz, CDCl_3_) of a PLA prepared by polymerization of rac-LA with **2_FeK_.**


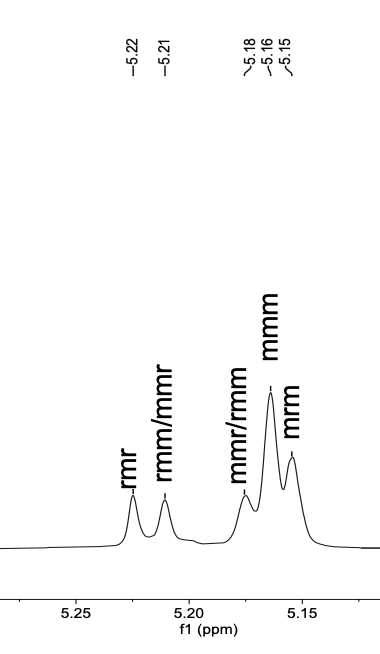


**Figure S19** Methine region of the homodecoupled ^1^H NMR spectrum (500 MHz, CDCl_3_) of a PLA prepared by polymerization of rac-LA with **2_FeNa_**.

### Polymerization activity


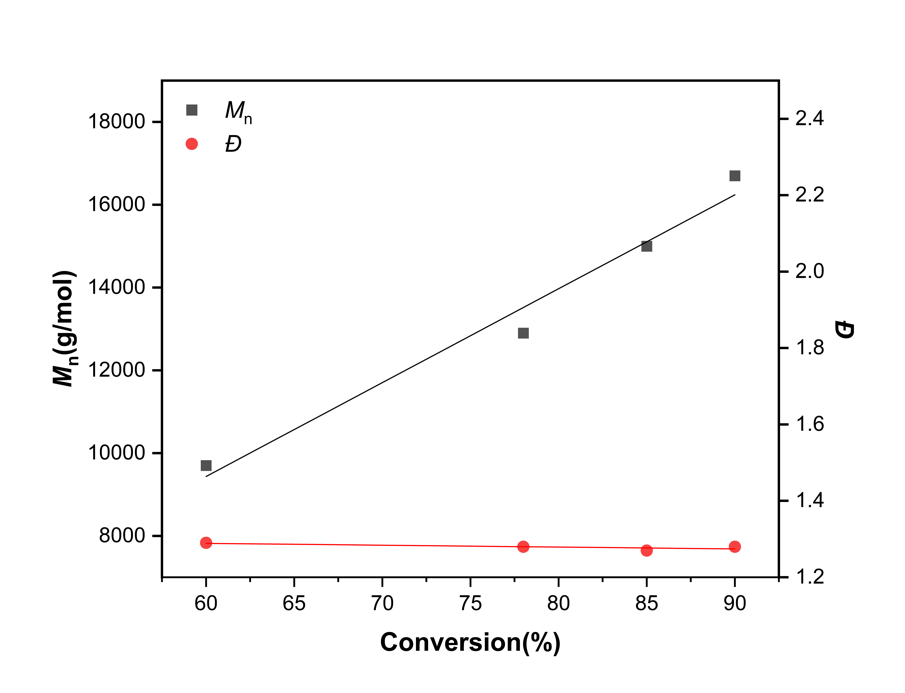


**Figure S20** Plot of M_n_ and Đ (vs polystyrene standards) as a function of monomer conversion (^1^H NMR) for the polymerization of rac-LA using **2_FeK_** ([rac-LA]_0_/[Cat.] = 200, toluene, RT).


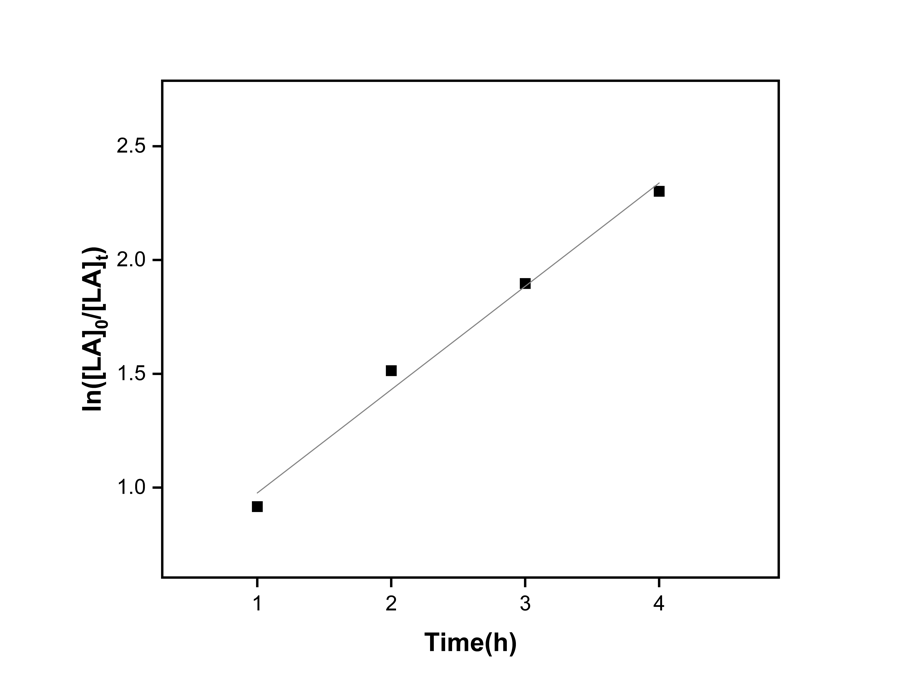


**Figure S21** Semilogarithmic plot of rac-LA conversion vs time using **2_FeK_** ([rac-LA]_0_/[Cat.] = 200, toluene, RT).

*
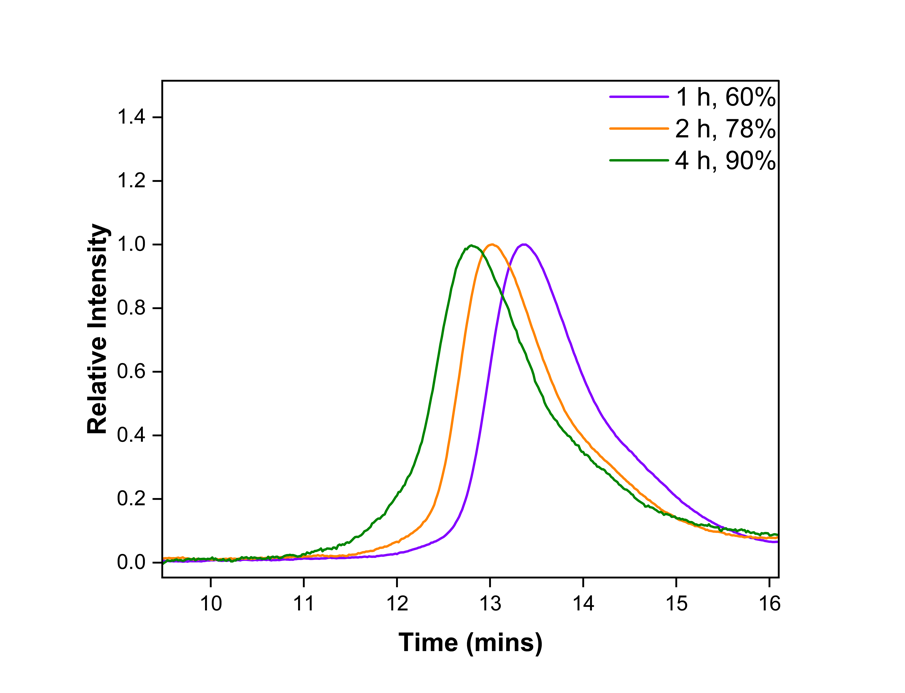
*


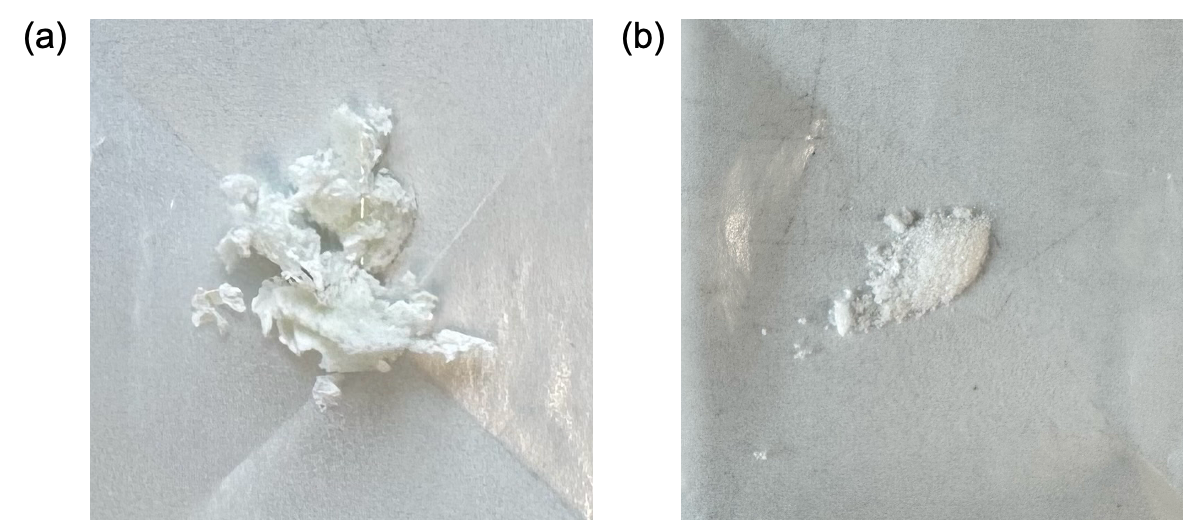
**Figure S22** SEC-RI traces of rac-LA polymerization using **2_FeK_** ([rac-LA]_0_/[Cat.] = 200, toluene, RT) over time.

**Figure S23** (a) PLA obtained with **1_CuK_** (M_n,exp_ = 22000 g/mol); (b) PLA obtained with **1_ZnK_** (M_n,exp_ = 3800 g/mol).

**Table S2.** Chain extension experiment of **2_FeK_**.^[a]^


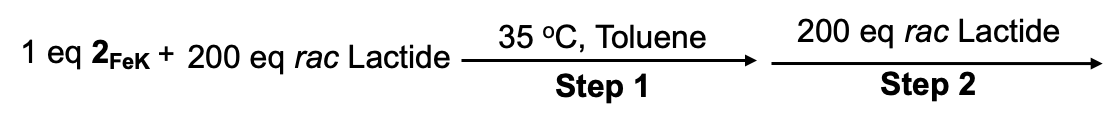


| **Entry** | **Conv.** ^[b]^ | ***M*_n,th_**^[c]^  **(g/mol)** | ***M*_n,exp_**^[d]^  **(g/mol)** | ***Đ***^[d]^ |
| --- | --- | --- | --- | --- |
| Step 1 | 93% | 13500 | 14000 | 1.28 |
| Step 2 | 93% | 27000^[e]^ | 20000 | 1.15 |

[a] Step 1 was performed with [LA]/[Cat] = 200/1, [LA]_0_ = 1 M at 35 ^o^C in toluene, after 4 h 200. eq of *rac* LA was added to the solution for another 4 h. [b] Determined by the integration of the ^1^H NMR methine resonances of LA and PLA. [c] *M*_n,th_ of polymers calculated from the monomer conversion: ([([LA]_0_/[Cat]) × 144.14 × conversion]/2[Metal]) + MW of end group. [d] *M*_n,exp_ and *Đ* determined by SEC-RI measurements in THF using polystyrene standards and *M*_n,exp_ corrected by the Mark-Houwink parameter for PLA (0.58). [e] *M*_n,th_ of polymers calculated from the monomer conversion: ([([LA]_0_/[Cat]) × 144.14 × (conversion Step1+ conversion Step2]/2[Metal]) + MW of end group.

### Recyclability of 2_FeK_.

**Table S3. Recycling tests of 2_FeK_ in ROP of rac-LA**.^[a]^

| **Entry** | **Conv.** ^[b]^ | ***M*_n,th_**^[c]^  **(g/mol)** | ***M*_n,exp_**^[d]^  **(g/mol)** | ***Đ***^[d]^ |
| --- | --- | --- | --- | --- |
| Cycle 1 | 100% | 7300 | 5400 | 1.26 |
| Cycle 2 | 95% | 6900 | 7400 | 1.17 |
| Cycle 3 | 93% | 6800 | 11000 | 1.39 |
| Cycle 4 | 75% | 5500 | 24000 | 1.16 |

[a] Each cycle was performed with [LA]/[Cat] = 100/1, [LA]_0_ = 1 M at 35 ^o^C in C_6_D_6_, after 2 h, catalyst was recycled by removed the solvent to performed next cycle. [b] Determined by the integration of the ^1^H NMR methine resonances of LA and PLA. [c] *M*_n,th_ of polymers calculated from the monomer conversion: ([([LA]_0_/[Cat]) × 144.14 × conversion]/2[Metal]) + MW of end group. [d] *M*_n,exp_ and *Đ* determined by SEC-RI measurements in THF using polystyrene standards and *M*_n,exp_ corrected by the Mark-Houwink parameter for PLA (0.58).

### Thermal property of PLA.

**Table S 4. Thermal analyses of PLA obtained in this study.** ^[a]^

| **Entry** | **Cat.** | ***M*_n,exp_^[b]^(g/mol)** | ***Đ*^[b]^** | ***T*_-5%_ (^o^C)** ^[c]^ | ***T*_g_ (^o^C)** ^[c]^ |
| --- | --- | --- | --- | --- | --- |
| 1 | **1_FeNa_** | 1600 | 2.24 | 185 | 41 |
| 2 | **1_CuK_** | 22000 | 1.54 | 250 | 52 |
| 3 | **1_ZnK_** | 3800 | 1.16 | 188 | 42 |

[a] All reactions performed with [LA]_0_ = 1 M at room temperature in toluene. [b] *M*_n,exp_ and *Đ* determined by SEC-RI measurements in THF using polystyrene standards and corrected by the Mark-Houwink parameter for PLA (0.58). [c] *T*_g_ of polymer determined by DSC on the second heating cycle (10 ^o^C/min, N_2_ flow). *T*_-5%_ of the polymer is determined by TGA (10 ^o^C/min, N_2_ flow).

The measured glass transition temperatures (*T*_g_, 41–52 °C) and temperatures at 5% weight loss (*T*_-5%_, 185–250 °C) are consistent with established trends for PLA. Specifically, *T*_g_ increases with *M*_n_ in accordance with the Fox–Flory relationship, while *T*_-5%_ falls within the ranges reported under comparable conditions.^[[8]](#endnote-8),^ ^[[9]](#endnote-9)^

**References**

1. I. Barakat, P. Dubois, R. Jérôme, P. Teyssié, Macromolecular Engineering of Polylactones and Polylactides. X. Selective End-Functionalization of Poly(D,L)-Lactide, *J. Polym. Sci., Part A: Polym. Chem.* **1993**, *31*, 505-514. [↑](#endnote-ref-1)
2. J. Baran, A. Duda, A. Kowalski, R. Szymanski, S. Penczek, Intermolecular Chain Transfer to Polymer With Chain Scission: General Treatment and Determination of k_p_/k_tr_ in L,L‐lactide Polymerization, *Macromol. Rapid Commun.* **1997**, *18*, 325-333. [↑](#endnote-ref-2)
3. Y. K. Gun’ko, U. Cristmann, V. G. Kelssler, Synthesis and Structure of the First Fe^II^ Heterometallic Alkoxide [(THF)NaFe(O*t*Bu)_3_]_2_ - a Possible Precursor for New Materials, *Eur. J. Inorg. Chem.* **2002**, 1029-1031. [↑](#endnote-ref-3)
4. A. A. Isah, O. Ohiro, L. Li, Y. Nasiru, K. C. Szeto, P.-Y. Dugas, A. Benayad, A. De Mallmann, S. L. Scott, B. R. Goldsmith, M. Taoufik, Selective Catalytic Reduction of CO_2_ to CO by a Single-Site Heterobimetallic Iron–Potassium Complex Supported on Alumina, *ACS Catal.* **2024**, *14*, 2418-2428. [↑](#endnote-ref-4)
5. C. E. Anson, W. Klopper, J.-S. Li, L. Ponikiewski, A. Rothenberger, A Close Look at Short C-CH_3_ Potassium Contacts: Synthetic and Theoretical Investigations of [M_2_Co_2_(μ_3_-O*t*Bu)_2_(μ_2_-O*t*Bu)_4_(thf)_n_] (M = Na, K, Rb, thf = tetrahydrofuran), *Chem. Eur. J.* **2006**, *12*, 2032-2038. [↑](#endnote-ref-5)
6. R. Becker, J. Weiß, M. Winter, K. Merz, R. A. Fischer, New Heterometallic Copper Zinc Alkoxides: Synthesis, Structure Properties and Pyrolysis to Cu/ZnO Composites, *J. Organomet. Chem.* **2001**, *630*, 253-262. [↑](#endnote-ref-6)
7. A. P. Purdy, C. F. George, Synthesis and Structure of Tri-*tert*-Butoxyzincates*, Polyhedron* **1994**, *13*, 709-712. [↑](#endnote-ref-7)
8. J. R. Dorgan, J. Janzen, M. P. Clayton, S. B. Hait, D. M. Knauss, Melt Rheology of Variable L-Content Poly(Lactic Acid), *J. Rheol.* **2005**, *49*, 607-619. [↑](#endnote-ref-8)
9. S. Farah, D. G. Anderson, R. Langer, Physical and Mechanical Properties of PLA, and their Functions in Widespread Applications - A Comprehensive Review, *Advanced Drug Delivery Reviews,* **2016***, 107,* 367-392. [↑](#endnote-ref-9)
